# Emotional dysregulation in a sample of adolescent inpatients: clinical and psychopathological characterization

**DOI:** 10.1192/j.eurpsy.2025.1168

**Published:** 2025-08-26

**Authors:** G. L. Nuzzo, G. Longo, A. Cicolini, L. Orsolini, U. Volpe

**Affiliations:** 1 Unit of Clinical Psychiatry, Department of Clinical Neurosciences/DIMSC, Polytechnic University of Marche, Ancona, Italy

## Abstract

**Introduction:**

Emotional dysregulation is an unhealthy emotional response to stimuli and a common reason for adolescent hospitalization. Linked to disorders like depression, borderline personality disorder, childhood trauma, and eating disorders, understanding its underlying causes may improve outcomes and prevent relapse.

**Objectives:**

The aim of this study is to characterize emotional dysregulation among adolescent impatient.

**Methods:**

Our study involves inpatients (16-24 years) hospitalized at our Transitional Psychiatric ward in Ancona (Università Politecnica delle Marche, Italy). The used rating scale were: Temperament Evaluation in Memphis, Pisa and San Diego (TEMPS-M), Difficulties in Emotion Regulation Scale (DERS), Barratt Impulsiveness Scale-11 (BIS-11), Toronto Alexithymia Scale-20 (TAS-20), Aggression Questionnaire (AQ). Descriptive analysis, simple and multivariate linear regression analysis were conducted.

**Results:**

97 adolescent patients were admitted from February 2022 to March 2023. The mean age of the sample is 17.3±1.9. The mean score to the BPRS is 43.9±10.3. The 70.7% (n=70) of the sample are females and the 79.8% (n=79) lives with their parents. Disruptive, impulse-control, and conduct disorders are the prevalent disorders in the sample (32.3%, n=32). The 21.1% (n=21) was diagnosed with depression, the 13.2% (n=13) with bipolar disorder and the 9.1% (n=9) with psychosis. According to the DERS, patients with emotional dysregulation are the 82,2% (n=37) of the sample. The 25.3% (n=24) of the sample could be classified as alexithymic. The most represented temperaments in the sample are the dysthymic 24.4% (n=11) and cyclothymic 22.2% (n=10). The mean score of the DERS is 122.33±29.5, the mean score of the TAS-20 is 58.9±166 and the mean score of the AQ is 78.7±30.1, the mean score of the BIS-11 is 65.2±19.1. A simple linear regression between DERS and AQ (R=0.536, R^2^=0.287, F(1)=15.284, p<0.001), TAS-20 (R=0.502, R^2^=0.252, F(1)=12.819, p=0.001) and BIS-11 (R=0.534, R^2^=0.285, F(1)=15.128, p<0.001) was observed. A multivariate linear regression was observed between the DERS (R=0.917, R^2^=0.842, F(1)=25.708, p<0.001) and the subscale about physical aggressivity of the AQ (β=2.065, p=0.008), the dysthymic subscale of the TEMPS (β=1.87, p<0.001), the hostility subscale of the AQ (β=-3.321, p<0.001), the subscale about difficulty identifying feelings of the TAS-20 (β=1.598, p=0.001), the total score of the AQ (β=0.5, p=0.006) and the subscale about cognitive impulsivity of the BIS-11 (β=1.024, p=0.047).

**Conclusions:**

The results suggest a link between emotional dysregulation, impulsivity, aggression, and alexithymia. Notably, emotional dysregulation appears in those with a dysthymic temperament, marked by high aggression, difficulty identifying feelings, cognitive impulsivity and low hostility. Further research is needed to explore these findings and develop treatment strategies.

**Disclosure of Interest:**

None Declared

